# Development of a Large-Range XY-Compliant Micropositioning Stage with Laser-Based Sensing and Active Disturbance Rejection Control

**DOI:** 10.3390/s24020663

**Published:** 2024-01-20

**Authors:** Ashenafi Abrham Kassa, Bijan Shirinzadeh, Kim Sang Tran, Kai Zhong Lai, Yanling Tian, Yanding Qin, Huaxian Wei

**Affiliations:** 1 Robotics and Mechatronics Research Laboratory (RMRL), Department of Mechanical and Aerospace Engineering, Monash University, Melbourne, VIC 3800, Australia; ashenafi.kassa@monash.edu (A.A.K.);; 2 School of Engineering, University of Warwick, Coventry CV4 7AL, UK; 3 College of Artificial Intelligence, Nankai University, Tianjin 300350, China; 4 Department of Mechanical Engineering, College of Engineering, Shantou University, Shantou 515063, China

**Keywords:** micropositioning stage, compliant mechanism, active disturbance rejection control, laser interferometer-based measurement

## Abstract

This paper presents a novel design and control strategies for a parallel two degrees-of-freedom (DOF) flexure-based micropositioning stage for large-range manipulation applications. The motion-guiding beam utilizes a compound hybrid compliant prismatic joint (CHCPJ) composed of corrugated and leaf flexures, ensuring increased compliance in primary directions and optimal stress distribution with minimal longitudinal length. Additionally, a four-beam parallelogram compliant prismatic joint (4BPCPJ) is used to improve the motion decoupling performance by increasing the off-axis to primary stiffness ratio. The mechanism’s output compliance and dynamic characteristics are analyzed using the compliance matrix method and Lagrange approach, respectively. The accuracy of the analysis is verified through finite element analysis (FEA) simulation. In order to examine the mechanism performance, a laser interferometer-based experimental setup is established. In addition, a linear active disturbance rejection control (LADRC) is developed to enhance the motion quality. Experimental results illustrate that the mechanism has the capability to provide a range of 2.5 mm and a resolution of 0.4 μm in both the X and Y axes. Furthermore, the developed stage has improved trajectory tracking and disturbance rejection capabilities.

## 1. Introduction

In the present era of advanced engineering and scientific advancements, manipulating and observing objects at the nano and microscale levels have emerged as the fundamental building blocks for further discoveries and advancements. To realize this, compliant mechanisms have been extensively explored recently to generate sub-nano/micrometer motion quality. Compliant mechanisms provide displacement, force, or energy through the elastic deformation of its flexural hinges [1]. This reduces friction, backlash, wear, and the need for lubrication [2,3]. In addition, as such mechanisms are usually manufactured monolithically, the manufacturing cost, assembly error, and complexity are reduced considerably [4]. Due to their indispensable properties of precision and accuracy, compliant mechanisms have been researched for use in various fields, including the medical sector [5,6,7], space and aerospace industries [8,9], micro/nano manipulation [10,11,12,13,14], robotics [15,16,17,18], and energy harvesting [19].

Recently, to meet the demand for a large range of precise translational motion, various kinds of parallel XY mechanisms have been synthesized. In designing a large range of micropositioning stages, cross-coupling, larger footprint size, modeling complexity arising from stiffness non-linearity, complex control algorithms, minimized resolution, and low dynamic performance are the main challenges. A four-PP (P-prismatic) parallel mechanism with spatial supporting linkages was proposed to increase workspace and payload capacity in [20,21]. In [22,23], a similar approach was used by using passive rigid links to constrain the unwanted motion in the orthogonal direction. However, such approaches limit the stroke and increase manufacturing complexity. In [24], the same configuration was proposed with a modular design using four double parallelogram mechanisms. Due to the longer and thinner leaf flexure used, a low resonant frequency of 15 Hz was achieved. To further increase the range with the same approach, a triple-stage compound double parallelogram flexure approach was introduced in [25]. Although this mechanism helps to provide a large stroke with pure translational motion, it degrades the dynamic performance of the mechanism due to the unconstrained intermediate stage [26,27]. To eliminate this problem, a nested linkage mechanism was used in [28]. However, the first resonant frequencies appeared lower than 5 Hz, and the stage had a large footprint size. Unlike serial mechanisms, parallel mechanisms exhibit higher cross-coupling, leading to degraded motion accuracy and complex control problems. Towards this, redundant constraints are often used to minimize cross-coupling and parasitic motion, but they limit motion range and add complexity to modeling and manufacturing [29,30]. Particularly, the typical manufacturing methods of monolithic flexure-based mechanisms are applicable to planar mechanisms with simple structures. Thus, having a simple design configuration is preferred to minimize the cost and manufacturing process. Concerning this, a modular architecture design is implemented in [31] to improve the functionality of flexure-based positioning stages by manufacturing reconfigurable design modules separately. It is typically challenging to attain a large workspace, higher bandwidth, and enhanced compactness simultaneously, so a better mechanism design is often required to strike a balance among these conflicting performance parameters [32].

To ensure precise and accurate motion, a mechanism with an appropriate kinematic configuration and carefully chosen constraints is not enough. The presence of inevitable factors such as cross-coupling, stress stiffening, parameter variation, and external disturbances often necessitates the implementation of an effective control strategy to compensate for these limitations and enhance the motion accuracy and precision of the mechanism [33,34].

In order to address these challenges, various control strategies have been implemented to drive flexure-based mechanisms. To improve the motion accuracy of a one-DOF mechanism, a sliding mode control (SMC) based on a PID sliding surface with an adaptive fuzzy disturbance observer (AFDO) was employed in [35]. The chattering effect was attenuated as the implemented observer provided an approximate estimation of the disturbance. The same approach was used with a feedforward control in [20] to drive an XY mechanism. The inverse of a Bouc–Wen-based hysteresis model was used in the feedforward control to compensate for the non-linearity of the actuator. Similarly, a nonlinear disturbance observer (NDO) was integrated with SMC to control a large-range three-DOF micropositioning stage [36]. Moreover, H-infinity [37], enhanced model predictive control [38], disturbance observer-based repetitive control [39], adaptive sliding mode control [40], hysteresis-compensated model reference adaptive control [41], and others were implemented on various flexure-based mechanisms. In prior studies, sliding mode control has commonly been employed to control large-range mechanisms due to its simplicity and robustness. However, this method suffers from chattering, which hinders smooth motion and shortens the lifespan of the actuator. It is essential to note that the effectiveness of the control methods implemented in previous studies largely depends on the accuracy of the mechanism’s model. However, accurately characterizing compliant mechanisms is difficult due to their coupled kinematics and elastomechanical behaviors [42]. Moreover, stress stiffening and parameter uncertainties pose additional challenges in accurately capturing the system dynamics. Some studies have investigated the modeling of stress stiffening [43] and actuator hysteresis [44,45,46] to enhance controller performance. However, this is a demanding approach that is typically effective for dealing with deterministic variations. A more efficient and promising alternative is to integrate disturbance estimation techniques to address unmodeled dynamics and parameter uncertainties [47]. Towards this issue, active disturbance rejection control (ADRC) is being proposed in different studies to enhance motion accuracy in the presence of parameter variations and disturbances. This control method employs simple strategies and requires minimal model information. Recently, ADRC has been implemented in many studies to address the hysteresis problem of the piezoelectric actuator in flexure-based mechanisms [48,49]. However, limited research has been conducted to explore the potential application of ADRC in voice coil motor (VCM)-driven large-range flexure-based mechanisms.

To this end, this paper introduces a novel large-range two-DOF mechanism by utilizing a hybrid configuration incorporating both corrugated and leaf flexures. A corrugated flexure possesses higher compliance and reduced stress concentration compared to a straight segment of equal longitudinal length [50,51,52]. This allows the development of the stage with an improved workspace while maintaining a compact size. Besides, redundant leaf flexures are employed to minimize the cross-coupling and enhance the rotational stiffness. In addition, a LADRC controller is developed to enhance motion precision and robustness with limited model information and a simple control structure. Due to its capability of providing accurate measurements over an extended range, the laser interferometry-based sensing and measurement (LISM) approach is adopted in this paper [53]. The experimental investigation reveals that the proposed mechanism, coupled with LADRC, achieves significant tracking accuracy and disturbance rejection capabilities.

This paper is organized as follows: The mechanism design is presented in Section 2, followed by analytical modeling in Section 3. Section 4 describes the computational analysis conducted using FEA. Section 5 presents the experimental setup, system identification, and controller design. In Section 6, experimental results are presented. Finally, the conclusion is provided in Section 7.

## 2. Structural Design

**Figure 1:**
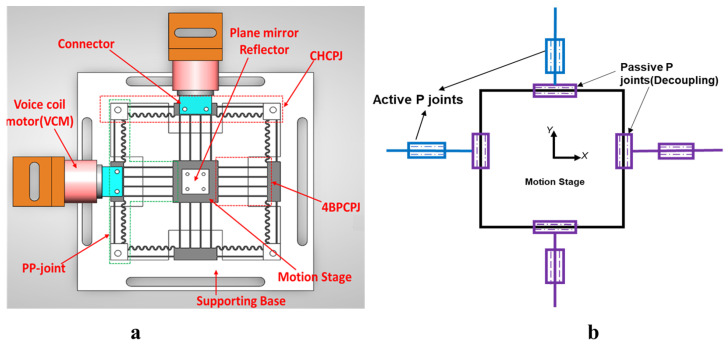
(**a**) Mechanism design; (**b**) 4PP kinematic configuration.

A four-PP symmetric parallel kinematic configuration is used to design the mechanism, as shown in Figure 1b. This design feature minimizes the parasitic in-plane rotation, improves the motion decoupling performance, and ensures consistent dynamic performance. The mechanism is formulated from four identical compliant legs. Each compliant leg is created by connecting two prismatic flexure modules in series. The first P joint is a compound hybrid compliant P joint (CHCPJ) made of corrugated and leaf flexures. A compound basic parallelogram has commonly been used to design prismatic joints in previous studies. This limits the stroke and requires a higher actuation force. A CHCPJ takes advantage of higher flexibility and the small strain range of the corrugated flexure [54]. Additionally, incorporating the corrugated flexure helps to distribute stress more evenly, resulting in a more robust and reliable design. However, the main drawback of the corrugated flexure is its higher compliance in the off-axial axes, which the CHCPJ addresses by combining it with the leaf flexure to maximize the ratio of off-axial stiffness to primary stiffness. Consequently, deformation along the degrees of constraint is minimized. This approach not only improves actuator isolation (input-decoupling) capability but also minimizes motion loss between the input stage and the output platform. A four-beam parallelogram P joint (4BPCPJ) based on a leaf flexure is the second joint used to transmit the input motion from the actuator to the moving platform as it has a higher longitudinal stiffness [55,56]. In addition, it serves as a prismatic joint for the motion in the orthogonal axis. Using redundant multiple beam flexures enhances the stiffness ratio without compromising the range [57,58]. This increased stiffness ratio can suppress the in-plane rotation of the moving platform and increase motion decoupling performance. Moreover, the utilization of the 4BPCPJ provides a key advantage in effectively distributing the applied compressive load, enhancing the mechanism’s robustness against buckling instability.

## 3. Analytical Modeling

The compliance matrix method and the energy-based Lagrange approach are chosen to analyze the output compliance and dynamic performance of the designed positioning stage. These analyses determine the relationship between the applied force and the displacements of the output platform, as well as the achievable bandwidth of the designed mechanism.

### 3.1. Output Compliance Modeling

The compliance matrix method is selected to determine the output compliance of the designed mechanism because of its conciseness and efficiency [59]. The in-plane compliance matrix at the output moving platform is formulated by transforming the compliance matrices of each flexure element from its local reference frame to the output reference frame.

With an applied load $F={\left[F_{x},F_{y},M_{z}\right]}^{T}$ and output motion $U={\left[x,y,\theta \right]}^{T}$, the in-plane compliance matrix of a flexure hinge in its local coordinate frame is formulated as follows:

(1) $$\left[\begin{array}{c} \delta_{x}\\ \delta_{y}\\ \theta_{z} \end{array}\right]=\left[\begin{array}{ccc} C_{1} & C_{2} & C_{3}\\ C_{4} & C5 & C_{6}\\ C_{7} & C_{8} & C_{9} \end{array}\right]\left[\begin{array}{c} F_{x}\\ F_{y}\\ M_{z} \end{array}\right]$$

The in-plane load–displacement relationship of a leaf flexure (Clf) and a unit semi-circular flexure (Ccu) fixed on one side with a constant thickness and center angle β(β=π) is derived in [60] using Castiglano’s displacement theorem as given in Table 1. The variables *b*, *l*, *t*, *R*, and *E* represent the width, length, thickness, radius, and Young’s modulus, respectively.

The compliance matrix of a given flexure element from its local reference frame “*i* ” can be transformed to the global coordinate frame “*j*” as follows:

(2) $$\begin{array}{c} C_{j}={\left[T\right]}^{T}{\left[R\right]}^{T}\left[C_{i}\right]\left[R\right]\left[T\right] \end{array}$$

where $R^{T}$ is the single axis rotation transformation matrix of the coordinate frame $O_{i}$ about another coordinate frame $O_{j}$, and *T* is the translation matrices from coordinate frame $O_{i}$ to $O_{j}$ expressed as:

(3) $$\begin{array}{c} T=\left[\begin{array}{ccc} 1 & 0 & 0\\ 0 & 1 & 0\\ y & -x & 1 \end{array}\right] \end{array}$$

where p=(x,y,0) is the position vector from reference frame $O_{i}$ to $O_{j}$.

Since the proposed mechanism is symmetric, only the compliance matrix of the upper section of Leg1 (Leg11) is calculated. The total output compliance can then be formulated by transforming the derived compliance matrix.

**Figure 2:**
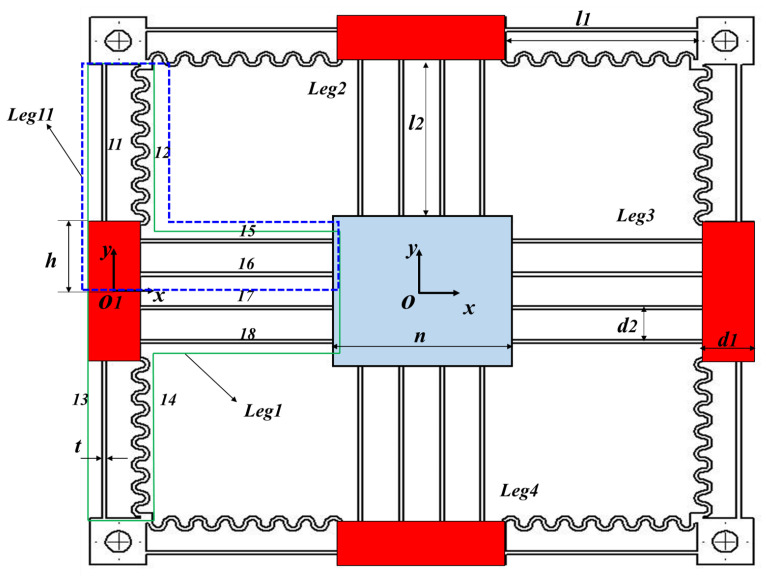
Mechanism schematic and design dimensions. Parameter dimensions are provided in Table 2.

**Figure 3:**
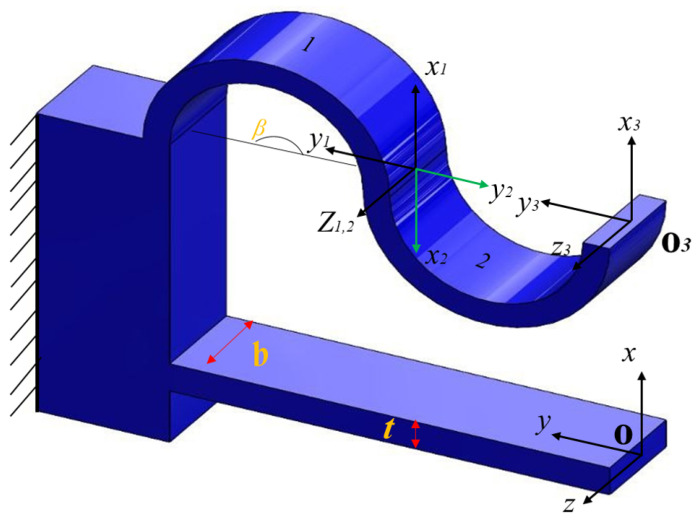
Hybrid flexure element with coordinate orientation in the local coordinate frame.

As shown in Figure 2, the compliance of the upper input hybrid compliant beam of Leg11 (11, 12) at coordinate $O_{1}$ can be calculated as:

(4) $$\begin{array}{c} C_{1}={\left[{\left[C_{11}\right]}^{-1}+{\left[C_{12}\right]}^{-1}\right]}^{-1} \end{array}$$

where $C_{11}$ is the compliance of the leaf beam at coordinate $O_{1}$, and $C_{12}$ is the compliance of the corrugated beam at coordinate $O_{1}$. The corrugated beam is composed of serially connected anti-symmetric corrugated units (cu), in which the total compliance can be calculated by adding each corrugated segment (cs).

(5) $$\begin{array}{c} \;C_{11}={\left[T_{p1}\right]}^{T}\left[C_{lf}\right]\left[T_{p1}\right] \end{array}$$

(6) $$\begin{array}{c} \;C_{12}={\left[T_{p2}\right]}^{T}\left[\sum_{i=1}^{7}{\left[T_{ri}\right]}^{T}\left[C_{cs}\right]\left[T_{ri}\right]\right]\left[T_{p2}\right] \end{array}$$

where T(p1) and T(p2) are the translation matrices with the position vector of $p1=[\frac{d1}{2},-h,0]$ and $p2=[\frac{-d1}{2},-h,0]$, respectively. T(ri) is the translation matrix with the position vector of ri=[0,−(7−i)(4R),0] where *i* is the number of corrugated segments. $C_{lf}$ is the compliance matrix of the leaf flexure beam from its tip, as given in Table 1. $C_{cs}$ is the compliance matrix of the corrugated segment at the tip of unit 2 ($x_{3}y_{3}z_{3}$), calculated by adding the compliance matrix of corrugated unit one and its anti-symmetric counterpart (unit two) shown in Figure 3, as follows:

(7) $$\begin{array}{c} C_{cs}={\left[T_{p3}\right]}^{T}[C_{cu}][T_{p3}]+{\left[T_{p3}\right]}^{T}{\left[R_{z}\left(\pi \right)\right]}^{T}[C_{cu}][R_{z}\left(\pi \right)][T_{p3}] \end{array}$$

where T(p3) is the translation matrix with the position vector of p3=[0,−2R,0]. The compliance matrix of beams 15 and 16 relative to frame O1 can be derived by rotating the compliance matrix of beam 11 along the Z-axis by π/2 and then translating by $p_{4}=\left[-\left(l_{2}+\frac{d1}{2}\right),-\left(\frac{d2}{2}+d2\right),0\right]$, and $p_{5}=\left[-\left(l_{2}+\frac{d1}{2}\right),-\left(\frac{d2}{2}\right),0\right]$, respectively, as follows:

(8) $$\begin{array}{c} C_{15}={\left[T_{p4}\right]}^{T}{\left[R_{z}\left(\pi /2\right)\right]}^{T}\left[C_{lf}\right]\left[R_{z}\left(\pi /2\right)\right]\left[T_{p4}\right] \end{array}$$

(9) $$\begin{array}{c} C_{16}={\left[T_{p5}\right]}^{T}{\left[R_{z}\left(\pi /2\right)\right]}^{T}\left[C_{lf}\right]\left[R_{z}\left(\pi /2\right)\right]\left[T_{p5}\right] \end{array}$$

Thus, the total compliance matrix of $C_{15}$, $C_{16}$, and $C_{1}$ at coordinate O1 can be calculated as:

(10) $$\begin{array}{c} C_{Leg11}={\left[{\left[C_{15}\right]}^{-1}+{\left[C_{16}\right]}^{-1}\right]}^{-1}+\left[C_{1}\right] \end{array}$$

Since the upper part of Leg1 (Leg11) is symmetric with the lower part (Leg12) about *X*-axis, the total compliance of Leg1 at coordinate O1 can be calculated as:

(11) $$\begin{array}{c} C_{Leg1}={\left[{\left[{\left[R_{x}\left(\pi \right)\right]}^{T}\left[C_{Leg11}\right]\left[R_{x}\left(\pi \right)\right]\right]}^{-1}+{\left[C_{Leg11}\right]}^{-1}\right]}^{-1} \end{array}$$

The total compliance of Leg1 at the global coordinate frame *O* ($C_{L1O}$) can be calculated by translating $C_{Leg1}$ along the position vector $p_{4}=\left[(l_{2}+\frac{d1+n}{2}),0,0\right]$ as follows:

(12) $$\begin{array}{c} C_{L1O}={\left[T_{P4}\right]}^{T}\left[C_{Leg1}\right]\left[T_{P4}\right] \end{array}$$

Using the developed compliance matrix $C_{L1O}$, the compliance matrix of Leg 2, 3, and 4 at the global coordinate frame *O* can be calculated as follows:

(13) $$\begin{array}{c} \;C_{L2O}=[R_{z}\left(-\pi /2\right){]}^{T}\left[C_{L10}\right][R_{z}\left(-\pi /2\right)] \end{array}$$

(14) $$\begin{array}{c} C_{L3O}={\left[R_{z}\left(-\pi \right)\right]}^{T}\left[C_{L10}\right]\left[R_{z}\left(-\pi \right)\right] \end{array}$$

(15) $$\begin{array}{c} \;C_{L4O}={\left[R_{z}\left(-3\pi /2\right)\right]}^{T}\left[C_{L10}\right]\left[R_{z}\left(-3\pi /2\right)\right] \end{array}$$

The overall output compliance of the proposed stage at the center of the moving platform can be calculated by the compliance parallel rule as follows:

(16) $$\begin{array}{c} C_{O}={\left[{\left[C_{L1O}\right]}^{-1}+{\left[C_{L2O}\right]}^{-1}+{\left[C_{L3O}\right]}^{-1}+{\left[C_{L40}\right]}^{-1}\right]}^{-1} \end{array}$$

It is important to note that the compliance model developed predicts the behavior of the designed stage, assuming only the linear stiffness curve. In the future, a more accurate non-linear spatial model will be developed to accurately capture the load–displacement relationship and payload capacity.

### 3.2. Dynamic Modeling

An energy-based approach of Lagrange’s equation is used to derive the natural frequencies of the mechanism. The output platform’s displacement ${\left(x,y\right)}^{T}$ is selected as the generalized coordinate. Assuming negligible motion loss, the input displacement is assumed to be equal to the end-effector’s displacement. As the mass distribution significantly affects the natural frequency, the effect of the moving coil of the actuator, connector, and mirror holder at the output platforms is taken into account. Lagrange’s equation is first formulated by calculating the total kinetic and potential energy. As the designed mechanism provides translational motion in the X and Y axes, only the first two working modes are considered in the analysis.

The total kinetic energy of the stage can be calculated as:

(17) $$\begin{array}{c} T=\frac{1}{2}\left[2M_{I}+M_{O}+M_{VS}+M_{VC}+M_{C}\right]\left({\dot{x}}^{2}+{\dot{y}}^{2}\right) \end{array}$$

where $M_{I}$ is the mass of the input rigid part (red color), $M_{O}$ is the mass of the output platform, $M_{VS}$ is the mass of a reflecting plane mirror holder for the LISM, $M_{VC}$ is the mass of the VCM moving coil, and $M_{C}$ is the mass of the connector of the VCM to the mechanism. In the analysis, the mass of the flexible beams is disregarded due to their insignificance compared to other rigid components.

The potential energy *V* in the generalized coordinate can be written as:

(18) $$\begin{array}{c} V=V_{x}+V_{y}=\frac{1}{2}k_{x}x^{2}+\frac{1}{2}k_{y}y^{2} \end{array}$$

where $k_{x}$ and $k_{y}$ are the output stiffness in *x* and *y* directions, respectively. Using Lagrange’s equation, the dynamics model can be established as:

(19) $$\begin{array}{cc} \frac{d}{dt}\left(\frac{\partial T}{\partial {\dot{q}}_{i}}\right)-\frac{\partial T}{\partial q_{i}}+\frac{\partial V}{\partial q_{i}} & =F_{vcm} \end{array}$$

where $q_{i}$ is a vector of the generalized coordinate (x,y), and $F_{vcm}$ is the input force from the VCM actuator. Considering the undamped free vibration, Equation (19) can be rewritten as:

(20) $$\begin{array}{c} M\ddot{q_{i}}+Kq_{i}=0 \end{array}$$

where *M* is the equivalent mass matrix in the x and y-axis given as Diag[$\left[2M_{I}+M_{O}+M_{VS}+M_{VC}+M_{C}\right],\left[2M_{I}+M_{O}+M_{VS}+M_{VC}+M_{C}\right]$] and *K* is the stiffness matrix given as Diag[$K_{x},K_{y}$]. From the theory of vibration, the eigenvalue problem can be written as:

(21) $$\begin{array}{c} \left(K-\lambda_{i}^{2}M\right)\Phi_{i}=0 \end{array}$$

where the eigenvector $\Phi_{i}$ represents the associated mode shapes with the eigenvalue $\lambda_{i}$. Then, the natural frequencies can be solved from the characteristics equation given by:

(22) $$\begin{array}{c} \left|k-\lambda_{i}^{2}M\right|=0 \end{array}$$

where the natural frequencies are given as:

(23) $$\begin{array}{c} f_{i}=\frac{\lambda_{i}}{2\pi } \end{array}$$

## 4. Computational Analysis

In order to validate the developed analytical models, FEA is performed using ANSYS 2020 R1 software. From the computational analysis, the output compliance, achievable workspace in the x and y directions, natural frequencies, and maximum stress are evaluated. The mechanism is manufactured using acrylonitrile butadiene styrene (ABS) with its properties given in Table 3.

**Figure 4:**
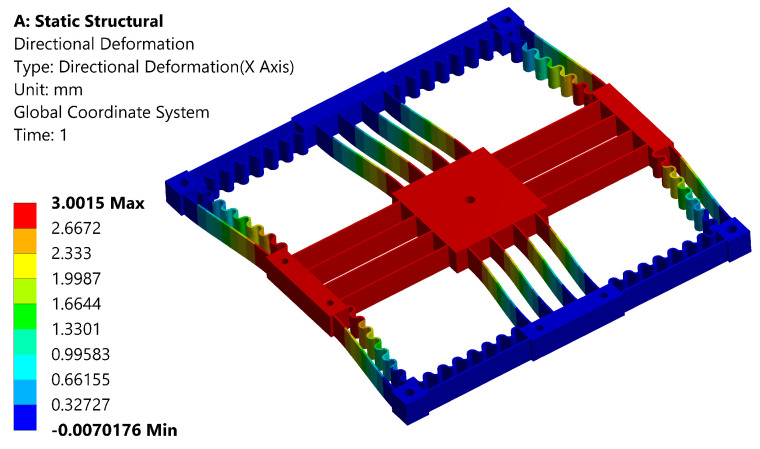
Maximum deformation analysis.

**Figure 5:**
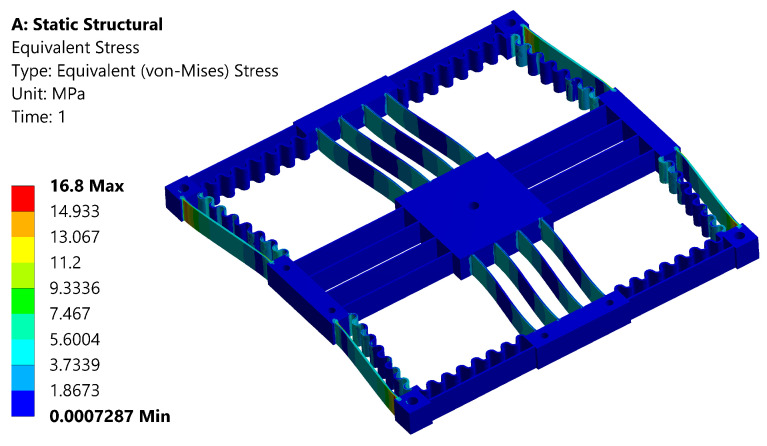
von Mises stress analysis of the positioning stage.

In order to evaluate the output compliance and maximum range of a mechanism, a series of input displacements were applied while imposing fixed constraints at the outer holes. The output compliance of the mechanism is determined by taking the ratio of displacement to the recorded reaction force. A force of 1 N is applied to the input compliant beam, which results in a displacement of 0.36 mm. The comparison between the analytical and FEA results is provided in Table 4, showing good agreement. The maximum displacement is determined to be 3mm with an equivalent von Mises stress of 16.8 Mpa, as shown in Figure 4 and Figure 5, respectively. The recorded maximum stress occurring in the leaf spring is less than the yield strength of ABS. The safety factor is determined to be more than 1.8, ensuring the mechanism operates in the elastic range with good repeatability. A linear buckling analysis was conducted to obtain an estimation of the critical load, prior to manufacturing and physical experiments. The analysis indicated that buckling typically occurs at approximately 113 N, a value significantly higher than the maximum actuation force of the VCM (87 N).

**Figure 6:**
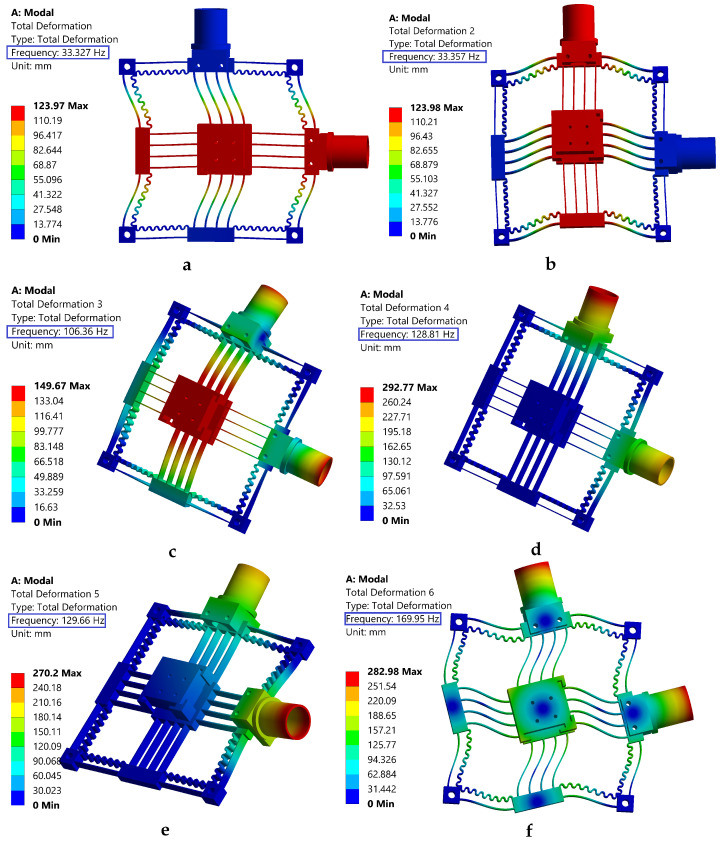
The first six mode shapes; (**a**,**b**,**f**) are in-plane modes; (**c**–**e**) are out-of-plane modes.

Modal analysis is carried out to determine the natural frequencies of the mechanism. Determining the dynamic performance is important, as the natural frequency determines the system’s overall response speed and bandwidth. In the analysis, the weight of the reflector mirror holder, the weight of the VCM connector, and the weight of the moving coil mass are taken into consideration. Figure 6 illustrates the first six natural frequencies and their respective mode shapes. The first two modes occur at 33.3 Hz and correspond to translational motion in the working direction along the *x* and *y* axes. The third mode (106 Hz) is the out-of-plane translational motion along the *z*-axis, indicating a higher out-of-plane stiffness and better payload capability. The sixth mode is due to the in-plane rotation that occurs at 170 Hz. From this, it can be inferred that the over-constrained leaf flexures maximize the rotational stiffness, effectively suppressing the parasitic in-plane rotation. By considering the FEA result as a benchmark, the error with the analytical result has a slight deviation, as shown in Table 4.

The third operating mode exhibits a frequency that exceeds three times that of the first two modes, which suggests that the proposed positioning stage possesses two degrees of freedom. This design ensures that higher frequency modes are less likely to be excited, thereby enhancing the in-plane translational capabilities. Considering the lower Young’s modulus of ABS, the mechanism has a competitive dynamic performance.

## 5. Experimental Setup

**Figure 7:**
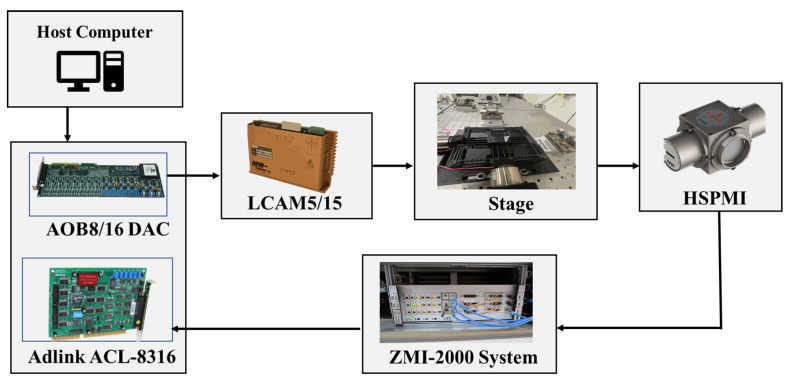
Experimental hardware setup and architecture.

**Figure 8:**
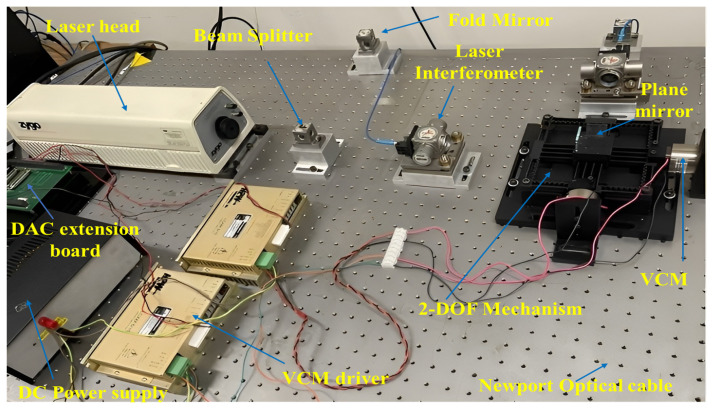
Experimental setup.

To examine the performance of the designed positioning stage and validate the theoretical model, experimental tests are conducted. The experimental hardware connection and setup are presented in Figure 7 and Figure 8, respectively. The mechanism’s dimension is 154 mm × 154 mm. Two voice coil motors from SUPT motion (VCAR0087-0062-00A) are utilized to actuate the mechanism. These VCMs can provide a stroke of up to 6.2 mm and a maximum force of 87 N. The VCMs are driven by two linear servo amplifiers (LCAM 5/15) from H2W, and the movement of the mechanism’s output platform is measured using a LISM setup from Zygo. Two high-stability plane mirror interferometers (HSPMI) are used to measure the translational motion of the plane mirrors at the end-effector in the X and Y axes. The optical signal of the measurement and the reference beam is transferred to the Zygo Motion Interferometer (ZMI-2000) system using fiber optic cables. The laser system’s reading is then transferred to the target PC via the Adlink ACL-8316 data acquisition card. The control signal from the target computer is converted into an analog signal using a 16-bit AOB8/16 Kontron DAC card and connected to the VCM driver. To minimize vibration issues, the overall setup is mounted on a Newport vibration-isolated optical table.

### 5.1. System Identification and Controller Design

#### 5.1.1. System Identification

**Figure 9:**
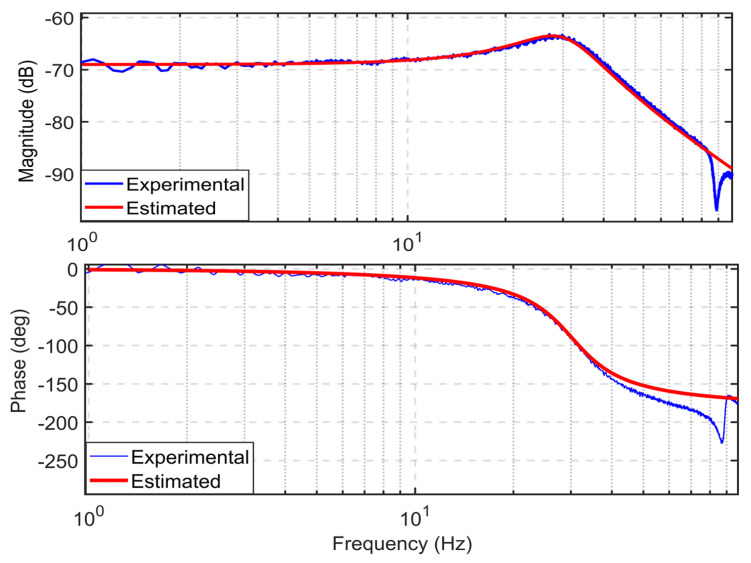
Open loop frequency response.

Experimental system identification is performed to determine the transfer function of the designed positioning stage. A chirp signal with an amplitude of 0.1 V and a 1-to-100 Hz frequency range is applied. The mechanism has a symmetric configuration, so the frequency response is performed along the *x*-axis. The open-loop frequency response of the estimated model and experimental data are shown in Figure 9. From the result, a second-order transfer function is identified as follows:

(24) $$\begin{array}{c} G\left(s\right)=\frac{13}{s^{2}+105s+3.6\times 10^{4}} \end{array}$$

The first natural frequency is identified as 30.2 Hz. The experimental result is a bit lower than the FEA with a deviation of 9.3%. This minimum discrepancy might arise from the weight of the plane mirror, as it was not considered in the FEA analysis.

#### 5.1.2. System Description

The designed flexure-based mechanism can be modeled as a second-order mass spring damper system. The relationship between the force generated by the VCM, and the input current is expressed in [61] as follows:

(25) $$\begin{array}{c} F_{vcm}=K_{f}i \end{array}$$

where $K_{f}$ is the force constant for the given VCM and *i* is the applied current. Using Kirchhoff’s voltage law, an equivalent circuit of a VCM is expressed as:

(26) $$\begin{array}{c} Ri+e+L\frac{di}{dt}=u \end{array}$$

(27) $$\begin{array}{c} \;e=K_{m}\frac{dx}{dt} \end{array}$$

where *u* is the input voltage, *e* is the back emf voltage which is proportional to the coil velocity with a constant $K_{m}$, and R and L are the coil resistance and inductance, respectively. Using the above equations, the overall system dynamics along the x-axis can be expressed as follows:

(28) $$\begin{array}{c} m\ddot{x}+c\dot{x}+kx=F_{vcm} \end{array}$$

where m is the equivalent mass of the stage along the x-axis, c is the damping coefficient, and k is the stiffness along the direction of the motion. Re-arranging and substituting Equations (25)–(27) into Equation (28) gives:

(29) $$\begin{array}{c} m\ddot{x}+c\dot{x}+kx=K_{f}\left[\frac{u-L\frac{di}{dt}-K_{m}\dot{x}}{R}\right] \end{array}$$

Re-arranging and introducing a disturbance d(t) in the above equation:

(30) $$\begin{array}{c} \ddot{x}=\left[a_{0}\frac{di}{dt}+a_{1}\dot{x}+a_{2}x+d\left(t\right)+\left(b-b_{0}\right)u\right]+b_{0}u \end{array}$$

where $a_{0}=\frac{-K_{f}L}{mR}$, $a_{1}=\frac{-(K_{f}K_{m}+cR)}{mR}$, $a_{2}=\frac{-K}{m}$, and $b_{0}\approx b\approx \frac{K_{f}}{mR}$. By introducing an additional state which is assumed to be differentiable, $f\left(t\right)=\left[a_{0}\frac{di}{dt}+a_{1}\dot{x}+a_{2}x+d\left(t\right)+\left(b-b_{0}\right)u\right]$, that lumps together the internal dynamics and the external disturbance d(t), the overall dynamics of the mechanism can be expressed in state space form as follows:

(31) $$\begin{array}{c} \left\{\begin{array}{cc} \dot{X} & =AX+Bu+E\dot{f}\\ y & =CX \end{array}\right. \end{array}$$

where $A=\left[\begin{array}{ccc} 0 & 1 & 0\\ 0 & 0 & 1\\ 0 & 0 & 0 \end{array}\right]$, $B=\left[\begin{array}{c} 0\\ b_{0}\\ 0 \end{array}\right]$, $C=\left[\begin{array}{ccc} 1 & 0 & 0 \end{array}\right]$, $E=\left[\begin{array}{c} 0\\ 0\\ 1 \end{array}\right]$

### 5.2. ADRC Controller Design

**Figure 10:**
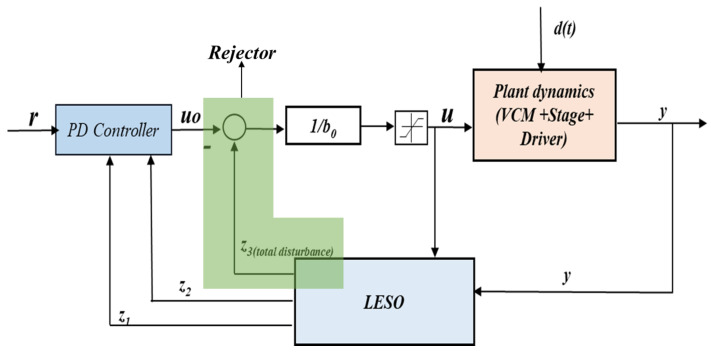
Active disturbance rejection control framework.

ADRC is a modern control strategy that overcomes the limitations of classical control techniques while retaining its simplicity [62,63]. It estimates and compensates for unknown internal dynamics and external disturbances, even when an accurate dynamical model of the system is not available. Initially developed as a nonlinear control strategy, ADRC was later modified into a linear version for easier tuning and stability analysis [64]. The main approach of ADRC is to lump together the external disturbance and the unknown internal dynamics as a generalized disturbance and then use an extended state observer (ESO) to estimate and compensate for it in real time, as shown in Figure 10. Hence, the system dynamics with parameter uncertainty and disturbance is converted into a simple double-integral plant after cancellation. The ranges of state and disturbance observers are explained in the literature. The linear ESO (LESO) based on the Luenberger observer is used in this study because of its simplicity [65].

LESO is formulated from the original plant dynamics by adding a correction term as follows:

(32) $$\begin{array}{c} \left\{\begin{array}{c} \dot{z}=Az+Bu+L\left(y-\hat{y}\right)\\ \hat{y}=Cz \end{array}\right. \end{array}$$

where $L={\left[\beta_{1}\;\beta_{2}\;\beta_{3}\right]}^{T}$ is the observer gain vector, and $z={\left[z_{1}\;z_{2}\;z_{3}\right]}^{T}$ is the estimated state vector. The primary goal is to choose suitable observer gains in order to ensure that the estimated states converge towards the actual states’ values.

From Equations (31) and (32), LESO can be expressed as:

(33) $$\begin{array}{c} \left\{\begin{array}{c} \dot{z_{1}}=z_{2}+\beta_{1}\left(y-z_{1}\right)\\ \dot{z_{2}}=z_{3}+\beta_{2}\left(y-z_{1}\right)+b_{0}u\\ \dot{z_{3}}=\beta_{3}\left(y-z_{1}\right) \end{array}\right. \end{array}$$

Considering the augmented state space expression given in Equations (31) and (33), ADRC control law is formulated as:

(34) $$\begin{array}{c} u=\frac{u_{0}-z_{3}}{b_{0}} \end{array}$$

(35) $$\begin{array}{c} u_{0}=k_{p}\left(r-z_{1}\right)-k_{d}\left(z_{2}\right) \end{array}$$

where r is the reference signal, $z_{3}$ is the generalized total disturbance, and $k_{p}\;\mathrm{and}\;k_{d}$ are the controller gains. Assuming LESO provides an accurate estimation of states such that $z_{3}\approx f\left(t\right)$, $z_{1}\approx x$, $z_{2}\approx \dot{x}$, Equation (30) can be written as:

(36) $$\begin{array}{c} \ddot{x}=k_{p}\left(r(t)-x\right)+k_{d}\left(\dot{x}\right) \end{array}$$

The transfer function can be formulated from Equation (36) as:

(37) $$\begin{array}{c} G_{cl}\left(s\right)=\frac{k_{p}}{s^{2}+k_{d}s+k_{p}} \end{array}$$

By choosing the closed loop poles to be at the same location $\left(-\omega_{c}\right)$ for a critically damped second order system, $k_{p}\;\mathrm{and}\;k_{d}$ can be determined from Equation (37) as:

(38) $$\begin{array}{c} k_{p}=\omega_{c}^{2}\;k_{d}=2\omega_{c} \end{array}$$

The observer gains can be identified from the characteristics polynomial of (A-LC) by placing all the poles at the same location ($-\omega_{0})$.

(39) $$\begin{array}{c} \lambda \left(s\right)={\left(s+\omega_{o}\right)}^{3}=s^{3}+\beta_{1}s^{2}+\beta_{2}s+\beta 3 \end{array}$$

From this, the observer gains can be determined as:

$$L={\left[3\omega_{0}\;3\omega_{0}^{2}\;\omega_{0}^{3}\right]}^{T}$$

## 6. Results

A range of experimental tests were conducted to assess the effectiveness of the LADRC control in trajectory tracking and disturbance rejection capability. The experimental results validate the absence of buckling or yielding phenomena, ensuring that the mechanism operates within a safe operational range. The positioning stage’s tracking ability, resolution, workspace, and robustness were evaluated through various experimental tests.

**Figure 11:**
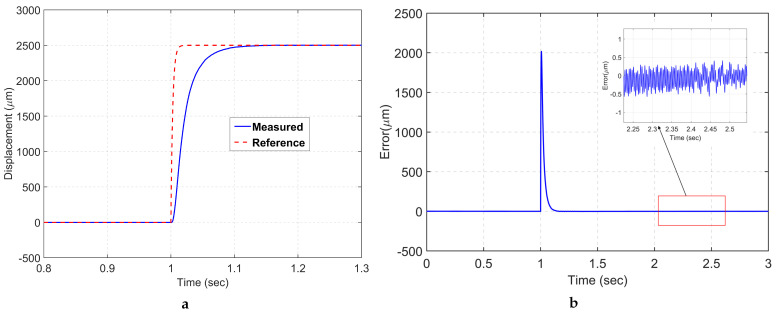
(**a**) Step response; (**b**) tracking error.

The designed mechanism is evaluated using a 2.5 mm pseudo-step command as the reference trajectory to assess its tracking performance and achievable workspace. Figure 11a shows that the LADRC exhibited a prompt response with no overshoot for the target positioning. The rise time and the 2% settling time are presented in Table 5. Additionally, the steady-state tracking error is kept within the range of ±0.5 μm, which is less than 0.02% of the total motion range, as demonstrated in Figure 11b.

**Figure 12:**
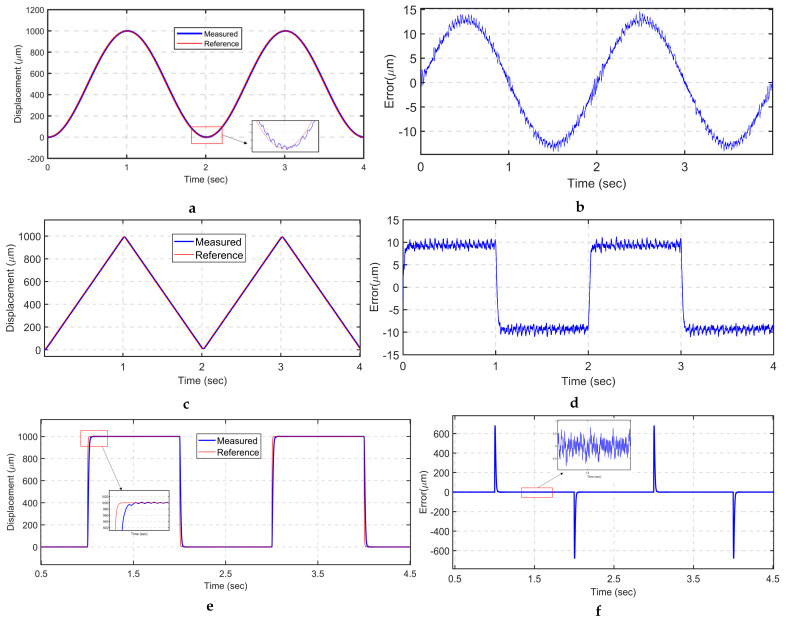
Periodic trajectory tracking: (**a**) sinusoidal trajectory; (**b**) tracking error of sinusoidal signal (**c**) triangular trajectory; (**d**) tracking error of triangular signal; (**e**) square trajectory tracking; (**f**) tracking error of square signal.

Several periodic trajectories are commanded to the VCM to assess the trajectory accuracy of the mechanism. A sinusoidal signal with an amplitude of 1 mm and a frequency of 0.5 Hz is applied to drive the VCM along the *x*-axis, as illustrated in Figure 12a. The root mean square error (RMSE) is approximately 9.15 μm, which corresponds to 0.915% of the total motion range. The maximum tracking error (MAXE) is restricted to 14.6 μm, which is 1.46% of the total stroke, as depicted in Figure 12b.

A triangular trajectory is commonly used in image scanning applications. Hence, a 1 mm peak-to-peak triangular signal with a frequency of half hertz is utilized, as shown in Figure 12c. The RMSE and MAXE are limited to 9.26 and 11.3 μm, respectively, accounting for 0.926% and 1.1% of the total range, respectively. It is worth noting that the highest error is found at the sharp turning corners. To overcome this issue, a smoothed triangular signal is implemented to ensure smooth trajectory tracking. Moreover, periodic square trajectories are used to evaluate the tracking performance of the positioning stage, as it is a commonly adopted trajectory scenario in object manipulation tasks. As shown in Figure 12f, LADRC tracks the command with a slight overshoot and minimum tracking error.

**Figure 13:**
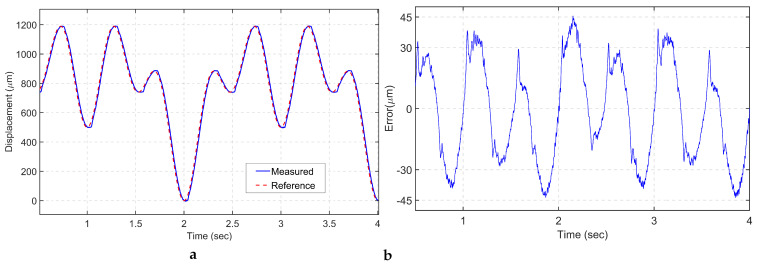
(**a**) Superimposed trajectory tracking; (**b**) tracking error.

To further assess the tracking performance of LADRC, a superimposed trajectory by combining different sinusoidal signals with varying frequency and amplitude is employed, as illustrated in Figure 13. The RMSE and MAXE errors are 24.3 and 45.5 μm, accounting for 2% and 3.79% of the total range, respectively. The system’s response to the dynamically varying reference command, especially with higher-frequency sinusoidal components, contributes to the observed higher tracking errors. This limitation is attributed to LESO, which effectively estimates only constant and slowly varying disturbances. In forthcoming studies, we aim to address this limitation by integrating adaptive disturbance estimators and improved control law techniques within the ADRC framework.

**Figure 14:**
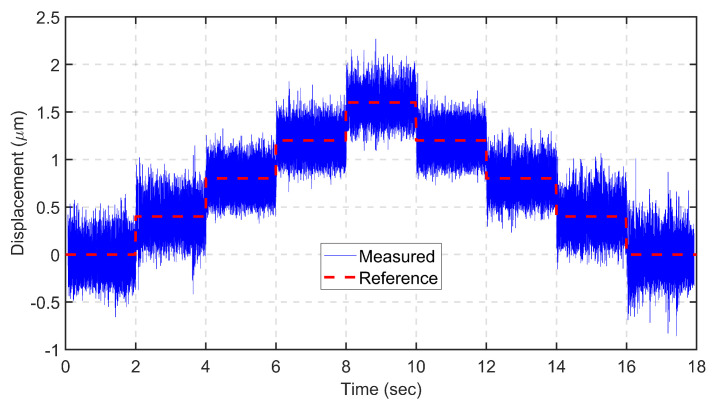
Closed loop resolution tracking.

To test the resolution of the designed mechanism, a consecutive multi-step signal with a time duration of 2 s and a step size of 0.4 μm is applied, as shown in Figure 14. Despite the mechanism being 3D printed and having a large range, a competitive resolution is achieved when compared with recently developed mechanisms.

**Figure 15:**
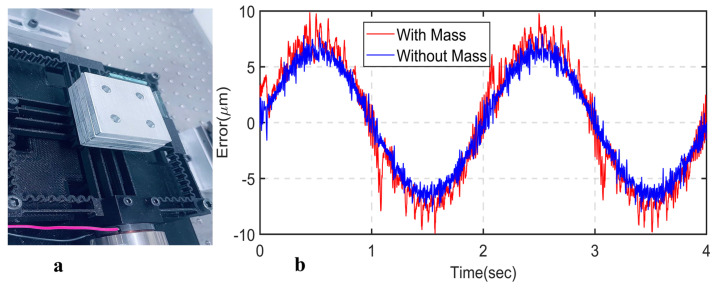
(**a**) Load mass on the end-effector; (**b**) sinusoidal tracking error with and without mass.

In order to evaluate the robustness of the designed control strategy in the presence of model uncertainties, metal bars are added to the positioning stage, as shown in Figure 15a. A 0.5 mm peak-to-peak sinusoidal trajectory with 0.5 Hz is tracked with and without the mass. The results demonstrate that LADRC can effectively track the signal with minimal error difference, as shown in Figure 15b. The improved estimation performance of the observer and real-time rejection of the lumped total disturbance ensures consistent performance in the presence of modeling uncertainty, as demonstrated in the experiment.

A comparison of the designed mechanism’s performance with recently manufactured positioning stages is presented in Table 6. As the manufacturing method and material properties play a significant role in the mechanism’s performance, the comparison is limited to additively manufactured stages. With regards to workspace, the proposed mechanism exhibits a greater range while maintaining enhanced compactness. In contrast, the design presented in reference [66] achieved improved natural frequency but at the cost of decreased range. It is important to note that the first natural frequency of the mechanisms discussed in references [67,68] is derived from finite element analysis and does not take into account certain factors such as the mass of the moving coil of the motor, motor connectors, and sensor frames. As a result, these designs are expected to exhibit a lower natural frequency. Overall, the designed mechanism is expected to provide an improved dynamic performance and enhanced motion repeatability if the stage is manufactured with a wire electrical discharge machining (WEDM).

## 7. Conclusions

This paper presented the development and control of a novel large-range two-DOF translational mechanism using hybrid compliant flexure joints. A four-PP kinematic configuration is utilized with redundant decoupling joints to suppress the in-plane rotation and minimize the cross-coupling with improved compactness. The stage’s output compliance and dynamic characteristics were formulated via the compliance matrix method and lumped parameter-based Lagrange’s equation. The developed model was verified using FEA. A high-resolution laser interferometer-based measurement was established to conduct the experimental analysis. A LADRC was employed to enhance the overall motion quality. The mechanism achieved precise motion tracking and disturbance rejection capability from the experimental results. The developed stage can provide a motion resolution of 0.4 μm and a range greater than 2.5 mm. This work provides important insights into the development of low-cost compliant stages as additive manufacturing advances. In future work, topologically optimized flexures with improved performance will be investigated to enhance the mechanism performance. In addition, more advanced controllers and observers will be integrated with the ADRC framework to further increase the motion quality.

## Figures and Tables

**Table 1:** Symmetric compliance matrix of leaf flexure and corrugated flexure.

C	Leaf Spring	Corrugated	C	Leaf Spring	Corrugated
$C_{1}$	$\frac{4l^{3}}{Ebt^{3}}$	$\frac{3R^{3}\left[6\beta -8sin\left(\beta \right)+sin\left(2\beta \right)\right]}{Ewt^{3}}$	$C_{6}$	0	$\frac{-12R^{2}\left(1-cos\left(\beta \right)\right)}{Ewt^{3}}$
$C_{2}$	0	$\frac{-24R^{3}sin^{4}\left(\frac{\beta }{2}\right)}{Ewt^{3}}$	$C_{7}$	$C_{3}$	$C_{3}$
$C_{3}$	$\frac{6l^{2}}{Et^{3}b}$	$\frac{12R^{2}\left(\beta -sin\left(\beta \right)\right)}{Ewt^{3}}$	$C_{8}$	0	$C_{6}$
$C_{4}$	0	$C_{2}$	$C_{9}$	$\frac{12l}{Et^{3}b}$	$\frac{12R\beta }{Ewt^{3}}$
$C_{5}$	$\frac{l}{Etb}$	$\frac{6R^{3}\left(\beta -sin\left(\beta \right)cos\left(\beta \right)\right)}{Ewt^{3}}$	−−	−−−−	−−−−

**Table 2:** Parameters of the XY flexure-based stage in millimetre.

Parameters	l1	l2	*t*	*n*	*h*	d1	d2	*R*	*b*
Value	42	45	1	40	20	12	9	2	10

**Table 3:** Material properties of ABS.

Young’s Modulus (E)	Tensile Yield Strength ($\sigma_{Y}$)	Density (ρ)	Poisson’s Ratio (ν)
2200 (MPa)	31 (MPa)	0.908 g/cm^3^	0.35

**Table 4:** Comparison of the output stiffness and first mode frequency.

Analysis	Analytical	Computational	Deviation (%)
First mode frequency	35.8 (Hz)	33.3 (Hz)	7.5
Output stiffness	2564 N/m	2777 N/m	−7.6

**Table 5:** 2.5 mm step command tracking performance specification.

Step Input	2% Settling Time (ms)	Rise Time (ms)	Percentage Overshoot
LADRC	88.2	43.5	0

**Table 6:** Performance comparison with additively manufactured XY mechanisms.

Stage	Dimension (mm^2^)	Range (mm)	First Nat. Freq (Hz)	Resolution (μm)
[66]	255 × 255	1 × 1	52.6	–
[67]	208 × 208	2 × 2	47.7	–
[68]	192 × 192	2 × 2	26.7	–
This work	154 × 154	2.5 × 2.5	30.2	0.4

## Data Availability

Data are contained within the article.

## Abbreviations

The following abbreviations are used in this manuscript:

CHCPJ	Compound hybrid compliant prismatic joint
4-BPCPJ	Four-beam parallelogram compliant prismatic joint
AFDO	Adaptive fuzzy disturbance observer
NDO	Nonlinear disturbance observer
LISM	Laser interferometry-based sensing and measurement
ABS	Acrylonitrile butadiene styrene
ESO	Extended state observer
WEDM	Wire electrical discharge machining
HSPMI	High-stability plane mirror interferometer
